# Effects of biochar produced at different pyrolysis temperatures on nitrogen transformation, microbial functional genes, and enzyme activities in saline soils

**DOI:** 10.3389/fmicb.2026.1801699

**Published:** 2026-03-16

**Authors:** Hui Zhou, Jiawei Guo, Jian Wang, Hu Liu

**Affiliations:** 1 Yinshanbeilu Grassland Eco-Hydrology National Observation and Research Station, China Institute of Water Resources and Hydropower Research, Beijing, China; 2 Yellow River Great Bend Region, Eco-environmental Change and Integrated Management Field Observation and Research Station of Inner Mongolia, Hohhot, China; 3 Institute of Water Resources for Pastoral Area Ministry of Water Resources, Hohhot, China; 4 College of Agronomy, Inner Mongolia Agricultural University, Hohhot, China

**Keywords:** biochar, functional genes, nitrogen mineralization, salinity level, soil enzymes

## Abstract

**Introduction:**

The application of biochar is generally considered to enhance soil nutrient availability and improve soil fertility, particularly in saline environments. However, the mechanisms by which biochar pyrolysis temperature influences nitrogen (N) availability and transformation in soils with varying degrees of salinity remain unclear.

**Methods:**

In this study, a laboratory incubation experiment was conducted to investigate the effects of biochar produced at different pyrolysis temperatures under three salinity levels (0.78, 1.92, and 2.79 dS m^−1^). Five treatments were applied: control (CK), conventional nitrogen application (N1), and N1 combined with 1% (w w^−1^) biochar produced at 300 °C (N1C1), 500 °C (N1C2), and 700 °C (N1C3). Soil microbial biomass, enzyme activities, nitrogen-cycling functional genes, and nitrogen mineralization processes were measured.

**Results:**

(1) Under low and moderate salinity, the N1C2 treatment significantly increased soil microbial biomass as well as urease and acid protease activities, whereas under high salinity, N1C3 performed better. Biochar addition also elevated the relative abundance of nitrogen-cycling functional genes, indicating enhanced net nitrogen transformation capacity. (2) Increasing salinity significantly suppressed nitrogen mineralization, while biochar application alleviated this inhibitory effect. Under low and moderate salinity conditions, the N1C2 treatment exhibited higher net nitrification and nitrogen mineralization capacities, whereas under high salinity conditions, the N1C3 treatment showed the most pronounced effect. (3) Soil microbial biomass and the activities of urease and acid protease were key factors influencing nitrogen mineralization across different salinity levels. With increasing salinity, the nitrogen mineralization pathway shifted from one dominated by aerobic mineralization/nitrification to more complex mechanisms involving facultative or anaerobic processes.

**Discussion:**

These findings provide valuable insights into nitrogen management in saline soils and underscore the importance of tailoring biochar applications according to pyrolysis temperature and salinity conditions.

## 1 Introduction

Soil salinization poses a significant threat to global agriculture and food security, with an estimated 1 billion hectares of land affected worldwide (Khondoker et al., 2023). China has approximately 36 million hectares of saline-alkali land, primarily distributed in arid and semi-arid regions (Li et al., 2014). Due to unique climatic conditions, soil properties, and anthropogenic influences, the northwestern arid regions of China exhibit particularly severe salinization, accounting for approximately 69% of the nation’s total saline-affected area (Yang et al., 2022). Salinity impairs nutrient balance, degrades soil physicochemical properties, intensifies osmotic stress, reduces crop yields, and ultimately diminishes economic returns (Huang et al., 2023). Given the continual reduction in arable land, the reclamation and utilization of saline-alkali soils are receiving increasing attention. Enhancing the agricultural productivity of such soils has become a strategic priority for ensuring global food security (Qadir et al., 2014). Rational reclamation of saline-alkali land requires a comprehensive understanding of the impact of salinity on the nitrogen (N) cycle, as N is not only a critical nutrient for plant growth and crop production but also a key factor in soil quality (Islam et al., 2024). Understanding how soil N cycling responds to salinity stress provides valuable insight for improving N use efficiency and mitigating the environmental losses of reactive nitrogen in saline environments.

Soil N transformation is central to the N cycle and involves biological nitrogen fixation, ammonification, nitrification, and denitrification, with organic N mineralization closely intertwined with these processes (Ding and Yu, 2025). In salt-affected soils, salinity is a dominant factor influencing N transformation. It imposes osmotic stress on plant roots, restricting N uptake (Hagemann, 2011). High salinity levels and poor soil properties in saline soils also shape distinct microbial communities (Zhang K. et al., 2019), which in turn alter microbially mediated N transformations (Duan et al., 2018). Furthermore, the dominant drivers of N transformation vary with salinity levels (Zhang W. W. et al., 2019), complicating N management strategies. Previous studies have demonstrated that saline soils typically contain lower levels of available N (Sun et al., 2021), and increasing salinity often inhibits N mineralization (Reddy and Crohn, 2014). Dendooven et al. (2010) found a negative correlation between nitrification rates and salinity levels. Conversely, Li and Huang (2008) observed that low salinity promoted ammonification and mineralization, whereas high salinity inhibited these processes. Zeng et al. (2013) reported that nitrification increased with salinity when EC₁_:_₅ was below 1.13 dS m^−1^ but declined beyond this threshold. These findings indicate that the response of soil N transformation to salinity gradients is complex and nonlinear.

Applying organic amendments is a promising strategy for modulating N release and improving N availability in salt-affected soils. In recent years, biochar has received considerable attention for ameliorating saline soils. Biochar is characterized by its high carbon content, chemical stability, porous structure, large specific surface area, and high cation exchange capacity, making it an effective soil amendment (Hematimatin et al., 2024; Zhang et al., 2021). Nitrogen is essential for crop production, and biochar has shown potential to reduce N loss and improve N use efficiency under salinity stress (An et al., 2023; Zhang et al., 2021). Studies have reported that biochar may enhance nitrification (Ulyett et al., 2013), suppress denitrification (Case et al., 2015), adsorb NH₄^+^-N to mitigate NH₃ and N₂O emissions (Sha et al., 2019), and increase the abundance of N-cycling genes (Zhang et al., 2021), thereby promoting plant N uptake. Thus, biochar is hypothesized to influence the mineralization of soil organic nitrogen (SON) in saline soils. However, the literature presents mixed findings—biochar has been reported to inhibit (Prayogo et al., 2013), stimulate (Ameloot et al., 2013a), or have no effect (Dempster et al., 2012) on N mineralization. For example, Maestrini et al. (2014) attributed enhanced mineralization to mineral N released from biochar, while Prayogo et al. (2013) observed suppression at biochar application rates of 0.5 and 2%, though the mechanisms remained unclear. Dempster et al. (2012) concluded that biochar had limited effects on the mineralization of low molecular weight dissolved organic N compounds in different agroecosystems. These findings suggest that biochar’s effect on SON mineralization is highly variable, depending on the biochar and soil types and their interactions.

Biochar properties are strongly influenced by pyrolysis temperature. Biochar produced at lower temperatures contains more labile organic matter and organic N, whereas high-temperature biochar is richer in aromatic compounds and possesses greater adsorptive capacity (Garbuz et al., 2021). Pantoja et al. (2024) found that low-temperature biochar had strong NH₃ adsorption capacity. A meta-analysis by Sha et al. (2019) indicated that biochar produced at around 400 °C was most effective at reducing NH₃ volatilization. Pyrolysis temperature affects not only N adsorption but also soil microbial communities, which play a key role in soil N transformation. High-temperature biochar typically has a greater specific surface area, porosity, pH, ash content, and carbon concentration, with lower toxicity to microbes (Cheng et al., 2017). However, excessive pyrolysis temperatures may inhibit microbial activity (Ameloot et al., 2013b). Thus, it is crucial to investigate how biochar produced at different temperatures influences soil N transformation and its associated microbial mechanisms (Zhang et al., 2021). Moreover, given that salinity significantly alters soil N cycling, it is plausible that the effects of biochar on N mineralization will vary along a salinity gradient. Therefore, a laboratory incubation experiment was conducted in this study to evaluate the effects of biochars produced at different pyrolysis temperatures on soil nitrogen mineralization under varying salinity levels, and to further elucidate the biological regulatory mechanisms underlying soil nitrogen transformation processes.

## 2 Materials and methods

### 2.1 Preparation of experimental samples

#### 2.1.1 Soil

Soil samples were collected from the saline-alkali soil remediation and improvement experimental site in Sunflower Town, Wuyuan County, located in the Hetao Irrigation District, Inner Mongolia, China (41°05′42″N, 108°20′42″E). This region has a temperate arid continental monsoon climate. The dominant salt type in the soil is NaCl, with a typical pH around 8. The degree of soil salinization varies considerably across the area, with electrical conductivity (EC) ranging from approximately 0.5–3 dS m^−1^. Prior to sowing in 2024, surface soil samples (0–20 cm) were collected for use in this study. After collection, the soil was thoroughly homogenized, air-dried indoors, and passed through a 2 mm sieve before being used in the incubation experiment. The basic physicochemical properties of the initial soil are presented in Table 1.

**Table 1:** Basic properties of tested soils.

Organic matter (g/kg)	Total N (g/kg)	Alkaline hydrolysis N (mg/kg)	Available P (mg/kg)	Available K (mg/kg)	EC (dS/m)	pH
8.26	0.91	33.26	26.32	152.39	0.77	8.2

#### 2.1.2 Biochar

Biochar was purchased from Haosen Environmental Protection Technology Co., Ltd. (Zhengzhou, China) and was produced from maize straw feedstock. Prior to pyrolysis, the raw material was washed, air-dried, and crushed, then further ground using a pulverizer for 5 min and stored for subsequent use. Naphthalene (analytical grade, purity >99%) was used in this study, with a molecular weight of 118.1 g mol^−1^ and a water solubility (Cs, 25 °C) of 30.3 g L^−1^. Biochars were produced using a continuous slow pyrolysis system. Before heating, nitrogen (N_2_) was introduced into the furnace at a flow rate of 0.1 L min^−1^ for approximately 10 min to establish an inert atmosphere. The samples were then heated at a rate of 10 °C min^−1^ to target temperatures of 300, 500, and 700 °C, respectively, and maintained at the designated temperature for 3 h. After pyrolysis, the samples were allowed to cool to room temperature under a continuous N_2_ atmosphere to prevent oxidation. The resulting biochar were designated as BC300, BC500, and BC700, according to their respective pyrolysis temperatures. Before use, all biochar samples were passed through a 2 mm sieve. The physicochemical properties of the biochar are presented in Table 2.

**Table 2:** Selected physicochemical characteristics of biochar produced at different pyrolysis temperatures.

Property	BC300	BC500	BC700
Surface area (m^2^/g)	1.9 ± 0.02 c	4.5 ± 0.02 b	7.3 ± 0.05 a
Pore volume (cm^3^/kg)	2.7 ± 0.01 c	6.7 ± 0.03 b	9.9 ± 0.05 a
EC (dS/m)	4.52 ± 0.05 c	5.21 ± 0.22 b	6.65 ± 0.96 a
CEC (cmol/kg)	14.5 ± 1.2 a	12.8 ± 0.9 b	8.0 ± 0.6 c
pH	7.8 ± 0.1 c	9.0 ± 0.1 b	10.0 ± 0.1 a
Productivity (%)	48.56 ± 1.6 a	33.25 ± 1.2 b	29.14 ± 0.9 c
C (%)	62.17 ± 0.3 c	65.36 ± 0.2 b	71.25 ± 0.1 a
H (%)	4.15 ± 0.05 a	3.33 ± 0.15 b	2.12 ± 0.09 c
N (%)	1.99 ± 0.12 a	0.52 ± 0.05 b	–
O (%)	21.02 ± 1.02 a	15.26 ± 0.82 b	8.68 ± 0.09 c
Mg (%)	0.25 ± 0.01 b	0.30 ± 0.02 a	0.31 ± 0.01 a
Al (%)	–	0.22 ± 0.01 a	0.23 ± 0.01 a
P (%)	0.51 ± 0.02 c	0.92 ± 0.03 b	1.53 ± 0.11 a
Ca (%)	0.82 ± 0.02 c	3.33 ± 0.01 b	4.98 ± 0.12 a
H/C	0.76 ± 0.01 a	0.51 ± 0.01 b	0.50 ± 0.02 b
O/C	0.20 ± 0.01 a	0.14 ± 0.03 b	0.08 ± 0.03 c
(O + N)/C	0.22 ± 0.01 a	0.14 ± 0.01 b	0.08 ± 0.01 c
TOC (g/kg)	417.79 ± 1.5 b	430.08 ± 1.3 b	518.93 ± 2.69 a
Total P (g/kg)	3.94 ± 0.09 a	2.57 ± 0.06 b	1.61 ± 0.03 c
Total K (g/kg)	54.15 ± 0.25 a	42.15 ± 0.69 b	38.25 ± 0.52 b

### 2.2 Experimental design

Three saline solutions with varying salinity levels were prepared using NaCl and distilled water, with electrical conductivity (EC) values of 7.15, 23.56, and 36.74 dS m^−1^, respectively. The test soil was leached with each solution using a funnel and subsequently oven-dried at 30 °C for 72 h. The dried soils were thoroughly homogenized and their EC values measured. This leaching-drying process was repeated until the soil EC reached levels corresponding to slight (0.78 dS m^−1^), moderate (1.92 dS m^−1^), and severe salinization (2.79 dS m^−1^), based on the classification criteria proposed by Shi (2011). The salinized soils were then air-dried at room temperature and stored until further use. Five treatments were established under soils with different salinity levels: a control (CK), a conventional nitrogen application (N1, equivalent to 300 kg N ha^−1^ based on the 0–20 cm soil layer), and three biochar treatments (N1C1, N1C2, and N1C3) combining conventional nitrogen with 1% (w/w) biochar produced at 300 °C, 500 °C, and 700 °C, respectively. The biochar application rate was optimized based on local recommendations. Each treatment was replicated three times under three salinity levels.

For incubation, 500 g of air-dried soil (sieved through a 1 mm mesh) was thoroughly mixed with the corresponding biochar and transferred into 1 L wide-mouth glass jars. The jars were placed in a dark, temperature-controlled incubator, with soil moisture adjusted to 80% of the water-holding capacity (WHC) according to Oustani et al. (2024). The pre-incubation lasted 7 days at 25 ± 1 °C to restore microbial activity. Thereafter, the soils were incubated for 90 days under the same temperature and moisture conditions. Throughout the incubation, jars were aerated for 1 h daily, and soil moisture was maintained by weighing and adjusting every 3 days to ensure it remained at 80% of WHC. Destructive sampling was conducted on days 1, 3, 14, 28, 42, 56, and 84 to determine soil physicochemical properties. Additionally, 5 g of soil was collected on days 14, 42, and 84, and stored at −20 °C for subsequent analysis of microbial functional genes related to the nitrogen cycle.

### 2.3 Measurement indices and methods

#### 2.3.1 Soil pH, ammonium nitrogen, nitrate nitrogen, total nitrogen, available phosphorus, and microbial biomass

Soil pH was measured using a pH meter (PHS−2F) following the method described by Pansu and Gautheyrou (2006). Total nitrogen (TN) was determined using the Kjeldahl digestion method (Lu, 2000). Available phosphorus (Olsen-P) was extracted with sodium bicarbonate and determined using the molybdenum-antimony anti-spectrophotometric method (Qi et al., 2024). Soil ammonium nitrogen (NH₄^+^-N) was extracted with 2 mol L^−1^ KCl and quantified using the indophenol blue colorimetric method, while nitrate nitrogen (NO₃^−^-N) was extracted with the same solution and determined using the dual-wavelength spectrophotometric method (Zheng et al., 2017). Soil microbial biomass was quantified using the chloroform fumigation method (Varma and Oelmüller, 2007).

#### 2.3.2 Soil enzyme activity test

Urease activity was expressed as the amount of NH₃^−^-N (μg) produced per gram of air-dried soil within 24 h (Kandeler and Gerber, 1988). Nitrate reductase activity was determined as the amount of NO₂^−^ (μmol) produced per gram of air-dried soil in 24 h (Barro et al., 1991). Nitrite reductase activity was calculated as the amount of NO₂^−^ (μmol) reduced per gram of air-dried soil in 24 h (Barro et al., 1991). Acid protease activity was expressed as the amount of tyrosine (μg) released per gram of air-dried soil over 24 h (Alef and Nannipieri, 1995).

#### 2.3.3 Determination of the relative abundance of NFGs

High-throughput analysis of microbial functional genes was conducted using the GeoChip 5.0 functional microarray platform, which includes four main steps: DNA extraction, labeling, hybridization, and image/data processing. GeoChip 5.0 is capable of detecting 57,000 oligonucleotide probes covering over 144,000 gene sequences from 373 gene families (Li et al., 2020).

Soil DNA was extracted from 0.50 g of soil per sample using the PowerSoil DNA Isolation Kit (MoBio, Carlsbad, CA, United States). For each treatment, soil sampling was performed in three biological replicates. The purity and concentration of extracted DNA were assessed using a spectrophotometer and the FLUOstar OPTIMA plate reader (BMG Labtech, Jena, Germany), respectively. Fluorescently labeled DNA was hybridized to the microarray, and the arrays were subsequently scanned to obtain hybridization images (Li et al., 2017). Each treatment was analyzed in triplicate using the microarray platform.

The DNA was labeled with Cy-3 fluorescent dye, purified using the QIAGEN QUICK Purification Kit (Roche NimbleGen Inc., United States), and dried at 45 °C for 45 min. Hybridization buffer was added, and the samples were denatured at 90 °C for 5 min, followed by pre-hybridization at 50 °C for 30 min. Hybridization was carried out on the MAUI hybridization system at 40 °C for 16 h. The microarrays were scanned at a wavelength of 633 nm. Hybridization images were processed and normalized using ImaGene 6.0 software, including inter-array normalization, removal of low-quality spots with a signal-to-noise ratio < 2.0, and log-transformation (natural logarithm) of signal intensity values (Zhang et al., 2017).

### 2.4 Data processing and analysis

Data were compiled and organized using Microsoft Excel 2019. Statistical analyses, including LSD and Duncan’s multiple range tests, were conducted using SPSS 24.0 (IBM, United States). Graphs and visualizations were generated with OriginPro 2021 (OriginLab Corporation, United States). Structural equation modeling (SEM) was performed using RStudio version 1.2 (RStudio Inc., United States) on the Windows NT platform. Statistical significance was determined at the level of *p* < 0.05.

## 3 Results

### 3.1 Variations in soil nitrogen, phosphorus, microbial biomass, and pH

**Figure 1:**
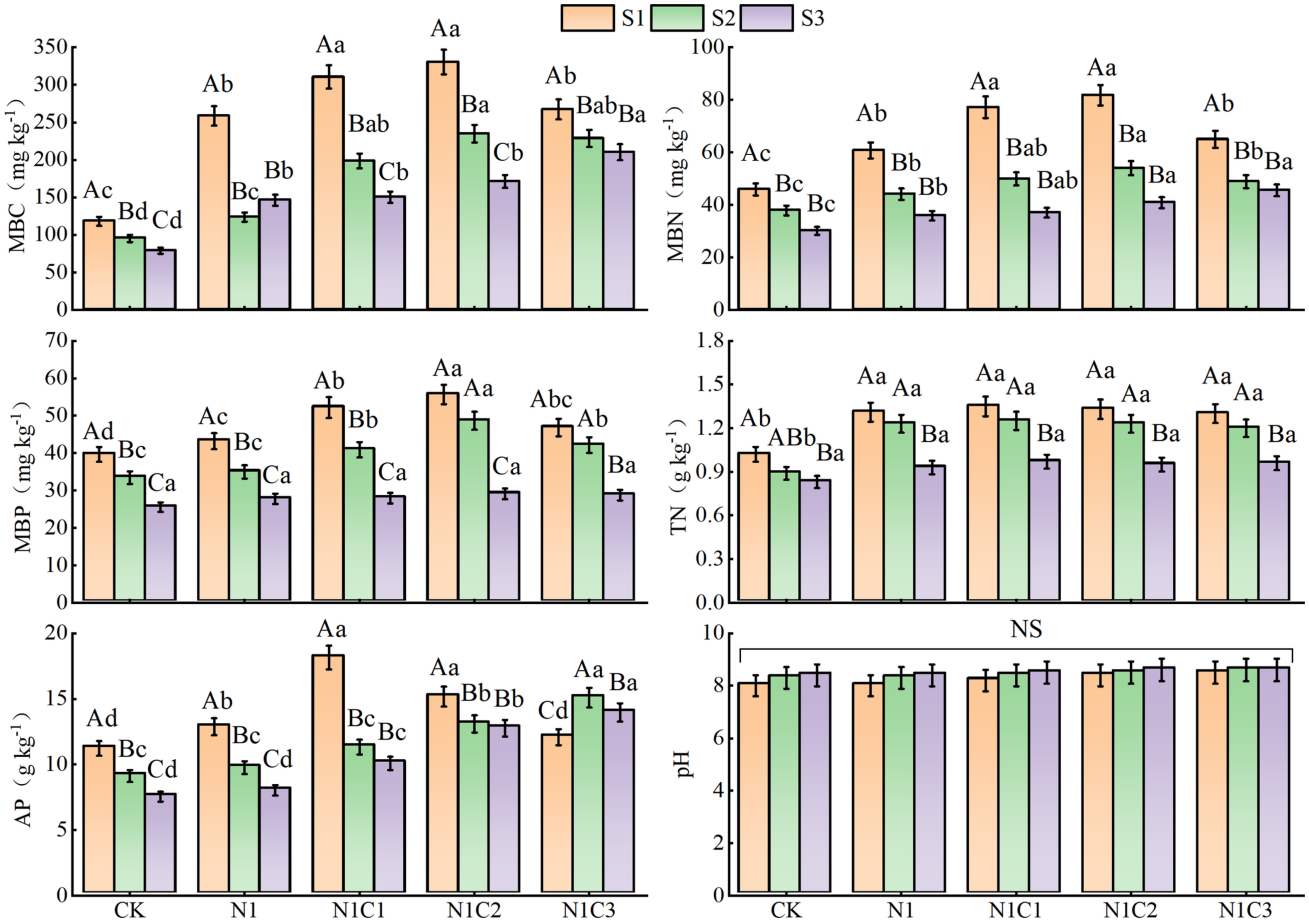
Variation patterns of soil MBC, MBN, MBP, TN, AP, and pH. Different uppercase and lowercase letters indicate significant differences between salinity levels and treatments, respectively, at the 0.05 significance level.

Overall, with increasing salinity levels, soil microbial biomass carbon (MBC), microbial biomass nitrogen (MBN), microbial biomass phosphorus (MBP), total nitrogen (TN), and available phosphorus (AP) showed a declining trend. However, the application of biochar improved these indicators to varying extents, effectively mitigating the inhibitory effects of salt stress on soil nutrient availability and microbial activity (Figure 1). Under the same salinity conditions, no significant differences in soil TN and pH were observed among the treatments. At salinity levels S1 and S2, the application of 300 °C and 500 °C pyrolyzed biochar resulted in higher MBC, MBN, and MBP contents, while soil AP was highest in treatments N1C1 and N1C3, respectively. Under the highest salinity level (S3), increasing pyrolysis temperature led to a gradual increase in MBC, MBN, and AP, whereas the effect on MBP was relatively minor.

### 3.2 Relative abundance of nitrogen functional genes (NFGs) and soil enzyme activities

**Figure 2:**
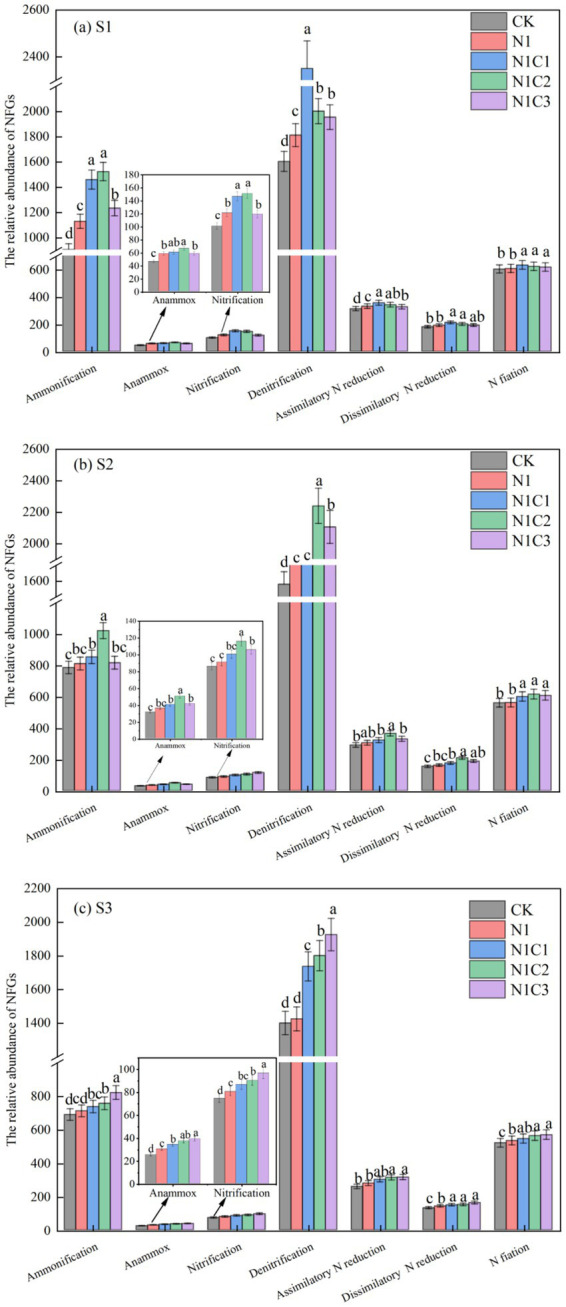
Relative abundance of nitrogen functional genes under different treatments at different salinity gradients: **(a)** S1, **(b)** S2, and **(c)** S3. Different uppercase and lowercase letters indicate significant differences between salinity levels and treatments, respectively, at the 0.05 significance level. The dashed line in the graph represents the mean value.

This study analyzed 15 nitrogen functional genes (NFGs) involved in seven metabolic pathways. Compared to the N1 treatment, biochar application increased the relative abundance of NFGs (Figure 2). Under the S1 salinity condition, the highest abundances of genes related to ammonification, anammox, and nitrification were observed in the N1C2 treatment, while denitrification, assimilatory nitrogen reduction, and dissimilatory nitrogen reduction genes were most abundant in the N1C1 treatment. Under S2 and S3 salinity conditions, the application of biochar pyrolyzed at 500 °C and 700 °C, respectively, resulted in the most significant increases in the relative abundance of nitrogen functional genes.

**Figure 3:**
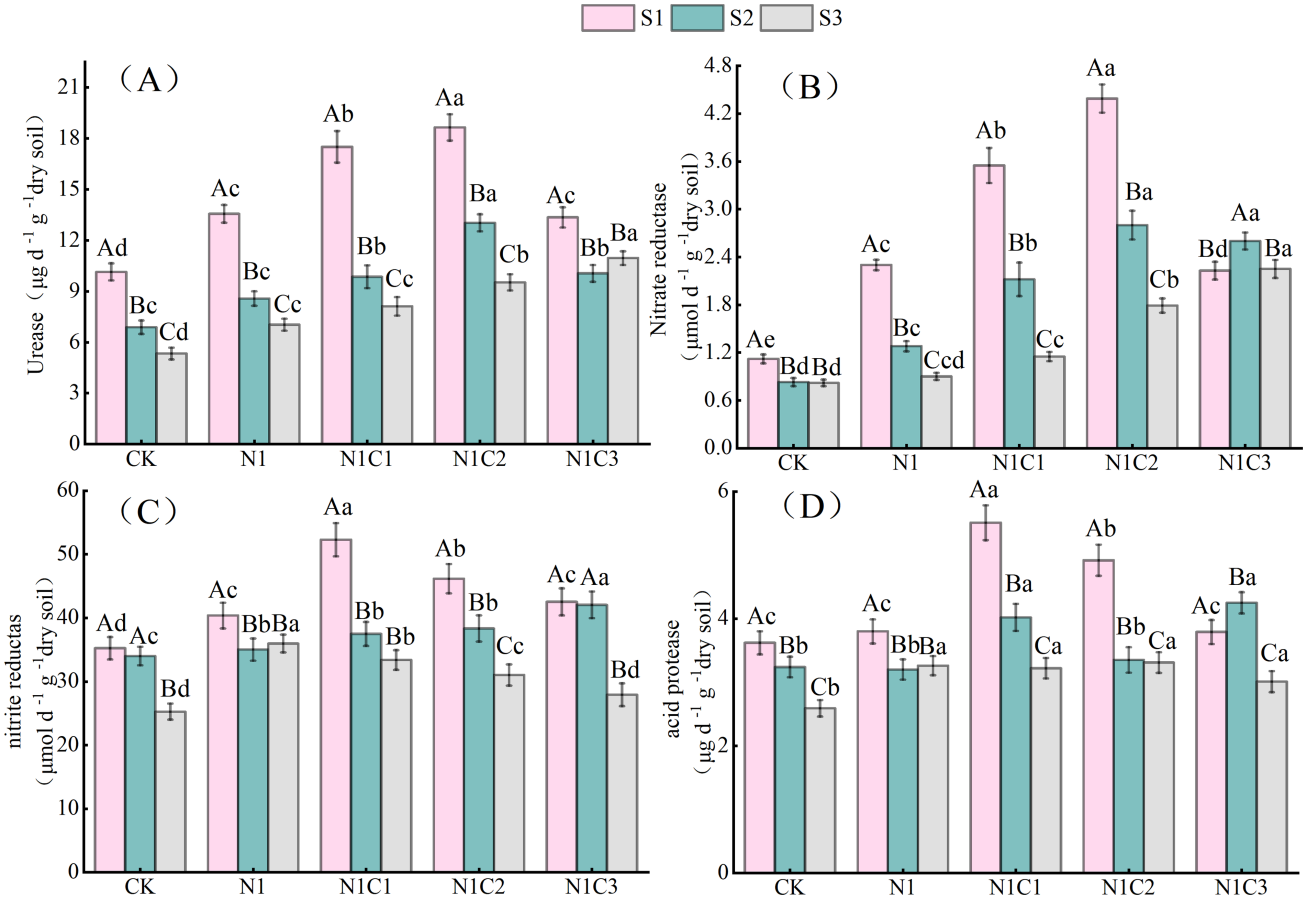
Differences in the activities of urease, nitrate reductase, nitrite reductase, and acid protease under different biochar treatments. The effects of biochar addition on urease, nitrate reductase, nitrite reductase, and acid protease activities are represented by **(A–D)** in the figure, respectively. Different uppercase and lowercase letters indicate significant differences between salinity levels and treatments, respectively, at the 0.05 significance level. The dashed line in the graph represents the mean value.

This study also found that soil salinity stress significantly reduced soil enzyme activities (Figure 3). Compared with the N1 treatment, biochar application enhanced soil enzyme activities to varying degrees. Under S1 and S2 salinity conditions, urease and acid protease activities first increased and then decreased with increasing pyrolysis temperature, reaching their highest levels in the N1C2 treatment. Nitrate reductase and nitrite reductase activities peaked in the N1C1 and N1C3 treatments, respectively. Under the highest salinity level (S3), urease and acid protease activities were highest in the N1C3 treatment, while nitrate reductase and nitrite reductase activities were greatest in the N1 treatment.

### 3.3 Soil nitrogen mineralization process

#### 3.3.1 Soil net ammonification

**Figure 4:**
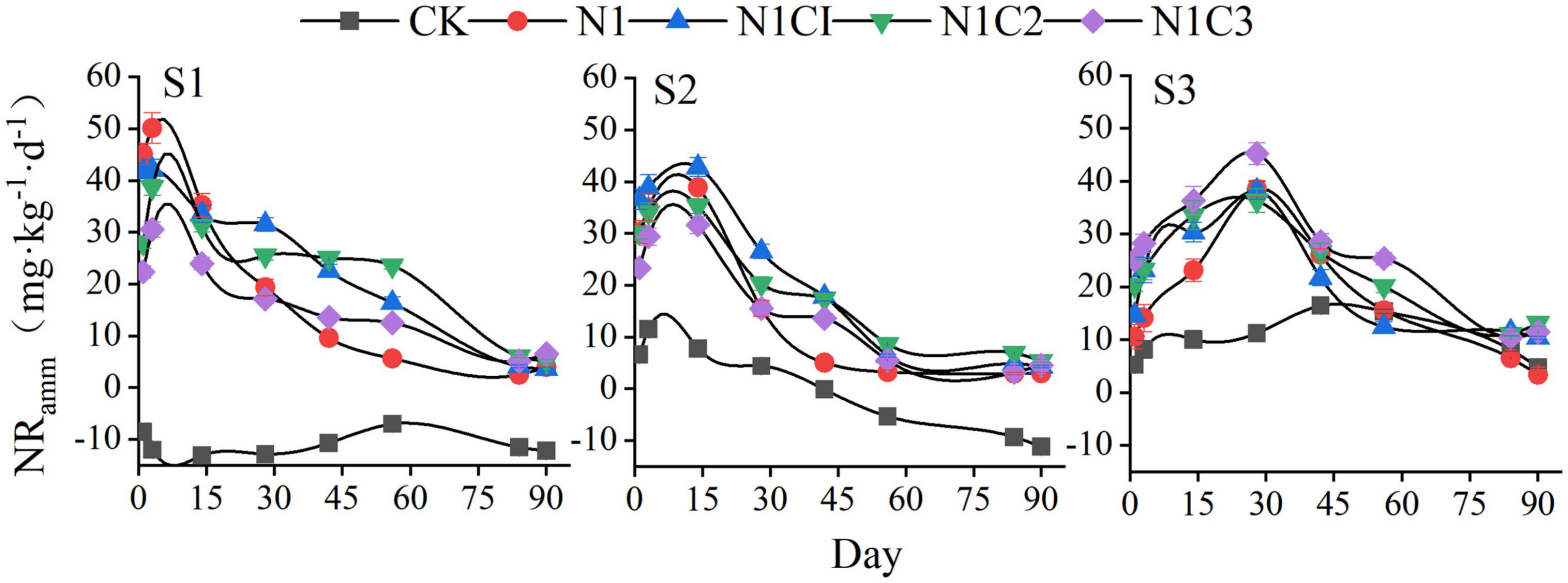
Soil net ammonification.

The pattern of soil ammonium nitrogen (NH₄^+^-N) release showed significant differences across different salinity levels and biochar types (Figure 4). Under the S1 salinity level, the net ammonification in the CK treatment remained consistently negative, while other treatments reached their peak net ammonification on day 3, with the N1 treatment exhibiting the highest value of 50.15 mg kg^−1^, which was significantly higher than other fertilization treatments by 19.01 to 64.10% (*p* < 0.05). Subsequently, net ammonification rapidly declined in all treatments, but the decline was delayed in biochar-amended soils, with the N1C2 treatment maintaining relatively higher ammonium content during the later stages of incubation. At the S2 salinity level, the ammonification peak for all treatments appeared around day 14, with the highest value observed in the N1C1 treatment at 42.81 mg kg^−1^, significantly exceeding other treatments by 10.11 to 35.26% (*p* < 0.05). Net ammonification tended to stabilize in all fertilized treatments around day 60. When soil salinity increased to the S3 level, the CK treatment’s ammonification turned positive, and peak ammonification occurred around day 30. With increasing biochar pyrolysis temperature, soil ammonification increased accordingly, with the N1C3 treatment reaching the highest peak of 45.26 mg kg^−1^, which was significantly higher than other fertilization treatments by 17.38 to 24.51%. All treatments showed a decline to relatively low ammonification levels at the end of incubation.

Two-way ANOVA results (Table 3) indicated that both soil salinity and biochar type had highly significant effects on soil net ammonification across different incubation periods. Except for day 90, the interaction between salinity and biochar type also had a highly significant influence on soil ammonification, suggesting that variations in net ammonification were closely related to both soil salinity and biochar pyrolysis temperature.

**Table 3:** Two-way ANOVA (*F*-values, *df, MS*) for soil net ammonification rate.

Cultural days (d)	Salinity (S)	Source of biochar (C)	C × N
1	790.21**, 2, 86.29	301.25**, 2, 31.25	30.25**, 4, 2.56
3	610.33**, 2, 65.33	312.44**, 2, 29.59	15.33**, 4, 1.41
14	84.26**, 2, 9.31	150.01**, 2, 164.33	36.58**, 4, 2.88
21	225.36**, 2, 20.56	15.73**, 2, 1.36	39.67**, 4, 4.15
28	455.15**, 2, 51.47	61.29**, 2, 5.98	19.98**, 4, 1.69
42	302.25**, 2, 29.56	133.38**, 2, 12.25	26.61**, 4, 2.14
56	456.61**, 2, 50.55	89.65**, 2, 8.26	13.33**, 4, 1.11
84	878.39**, 2, 91.23	57.89**, 2, 7.15	40.02**, 4, 4.23
90	68.17**, 2, 7.11	5.19**, 2, 0.77	2.27, 4, 0.36

*n* = 243, *p < 0.05, **p < 0.01.

#### 3.3.2 Soil net nitrification

**Figure 5:**
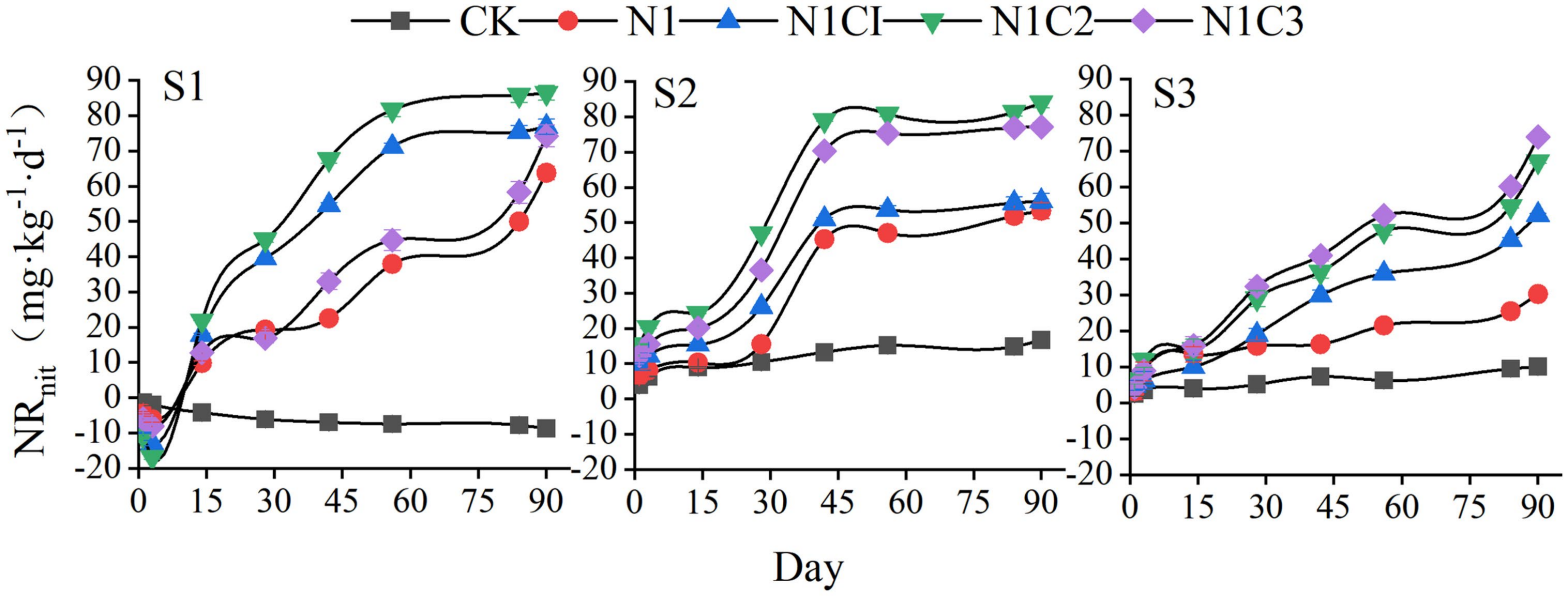
Soil net nitrification.

Soil salinity and biochar pyrolysis temperature had a notable influence on net nitrification during the incubation period (Figure 5). Under the S1 salinity condition, the net nitrification rate of the CK treatment remained negative throughout the incubation. In contrast, the fertilized treatments exhibited negative values during the initial 3 days, which subsequently turned positive. All fertilized treatments showed a gradual increase in net nitrification over time. The N1C1 and N1C2 treatments reached a relatively stable state around day 60, with the highest nitrification peak observed in the N1C2 treatment at 86.42 mg kg^−1^—12.45 to 35.50% higher than other fertilization treatments. Under the S2 salinity condition, net nitrification in the CK treatment turned positive. The time required to reach stabilization was shorter than under S1 conditions. The N1C2 treatment again showed the highest nitrification peak (83.98 mg kg^−1^), exceeding other treatments by 8.85 to 57.38%. Under the S3 salinity condition, the growth rate of net nitrification slowed compared to the S1 and S2 conditions. However, all fertilized treatments continued to show an upward trend in net nitrification by the end of the incubation period, with a clear pattern of increasing nitrification corresponding to higher biochar pyrolysis temperatures.

Two-way ANOVA results (Table 4) revealed that both soil salinity and biochar type had highly significant effects on soil net nitrification at different incubation stages. Except for days 84 and 90, their interaction also exerted a significant influence, indicating that soil nitrification dynamics were strongly regulated by both salinity levels and biochar pyrolysis temperatures throughout the incubation.

**Table 4:** Two-way ANOVA (*F*-values, *df*, *MS*) for soil net nitrification.

Cultural days	Salinity	Source of C	S × N
1	4283.55**, 2, 409.69	35.69**, 2, 2.94	145.33**, 4, 13.36
3	1502.33**, 2, 149.56	150.16**, 2, 13.33	80.25**, 4, 7.58
14	144.32**, 2, 15.54	598.36**, 2, 52.33	38.26**, 4, 3.26
21	57.69**, 2, 5.57	25.69**, 2, 2.14	56.33**, 4, 5.63
28	452.36**, 2, 47.25	188.59**, 2, 19.95	11.29**, 4, 1.30
42	400.69**, 2, 37.92	173.69**, 2, 15.54	10.03**, 4, 1.12
56	85.26**, 2, 9.33	45.61**, 2, 4.31	4.98**, 4, 0.64
84	25.36**, 2, 2.13	19.89**, 2, 1.57	3.59*, 4, 0.33
90	282.25**, 2, 31.59	67.37**, 2, 7.32	1.59, 4, 0.20

*n* = 243, *P < 0.05, **P < 0.01.

#### 3.3.3 Soil net nitrogen mineralization

**Figure 6:**
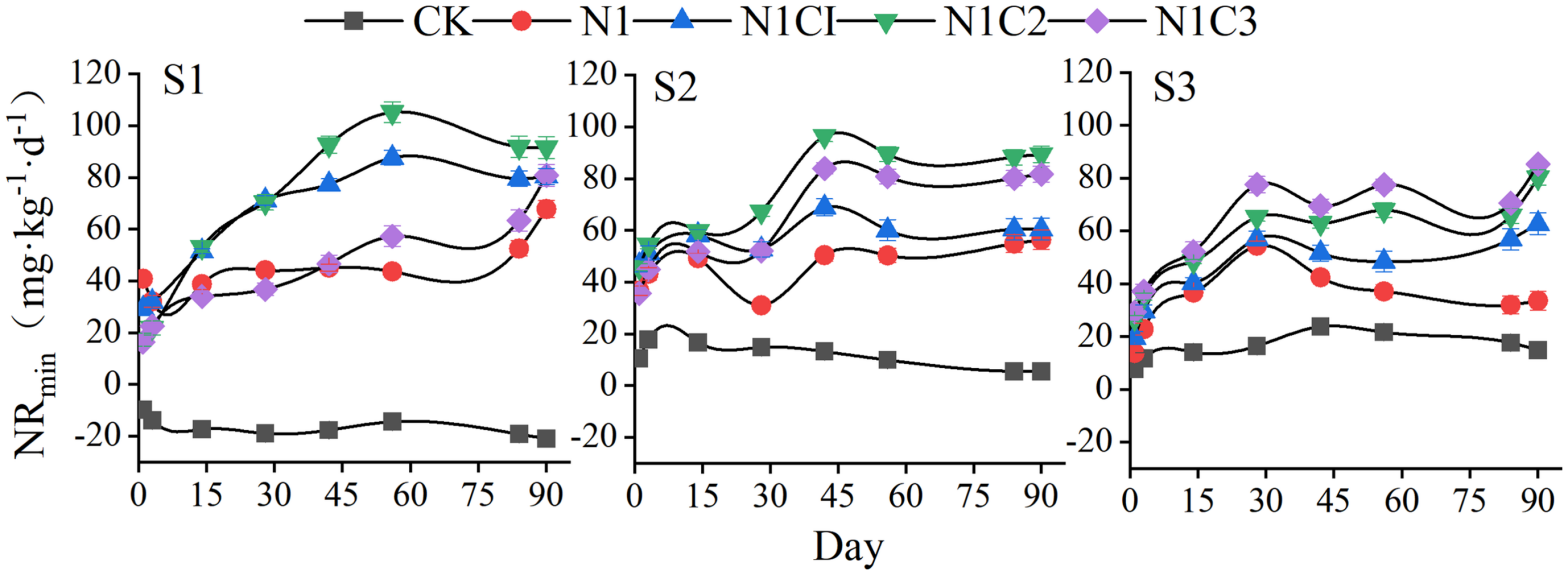
Soil net nitrogen mineralization.

As shown in Figure 6, under the S1 salinity condition, the net nitrogen mineralization in the CK treatment remained negative throughout the incubation period, continuously decreasing to −20.89 mg kg^−1^ by the end of incubation. In contrast, all fertilized treatments exhibited positive net mineralization values, generally increasing over time and reaching peak levels around day 56, with the N1C2 treatment showing the highest value—significantly exceeding other treatments by 14.56 to 55.25%. Under the S2 salinity condition, net mineralization in the CK treatment became positive. The mineralization values in all treatments fluctuated during incubation and generally stabilized by day 56, with the N1C2 treatment again displaying the highest value. Under the S3 salinity condition, net mineralization decreased across all treatments, accompanied by a reduced mineralization rate. However, net nitrogen mineralization increased progressively with rising biochar pyrolysis temperature during the incubation, and by the end of the experiment, the N1C3 treatment exhibited values 6.27–153.85% higher than other treatments.

Two-way ANOVA results (Table 5) demonstrated that both soil salinity and biochar pyrolysis temperature had highly significant effects on net nitrogen mineralization during the incubation period. Their interaction also had significant effects at all incubation stages except days 78 and 90. Based on *F*-values, biochar pyrolysis temperature emerged as the dominant factor influencing the nitrogen mineralization process.

**Table 5:** Two-way ANOVA (*F*-values, *df*, *MS*) for soil net nitrogen mineralization.

Cultural days	Salinity	Source of C	S × C
1	43.59**, 2, 4.31	358.70**, 2, 36.92	19.65**, 4, 21.35
3	49.25**, 2, 5.16	510.23**, 2, 48.36	8.98**, 4, 1.03
14	28.69**, 2, 2.74	333.25**, 2, 35.51	11.25**, 4, 1.16
21	30.16**, 2, 2.84	200.98**, 2, 19.58	9.33**, 4, 0.99
28	74.33**, 2, 6.89	219.56**, 2, 23.35	8.56**, 4, 0.75
42	125.65**, 2, 13.31	112.69**, 2, 10.66	3.99**, 4, 0.31
56	105.26**, 2, 11.02	142.36**, 2, 13.38	5.31**, 4, 0.39
84	67.89**, 2, 6.52	103.25**, 2, 9.98	1.26, 4, 0.11
90	33.25**, 2, 3.14	96.35**, 2, 11.55	1.36, 4, 0.13

*n* = 243, *P < 0.05, **P < 0.01.

### 3.4 Correlation and principal component analyses of soil environmental factors and nitrogen mineralization processes

**Figure 7:**
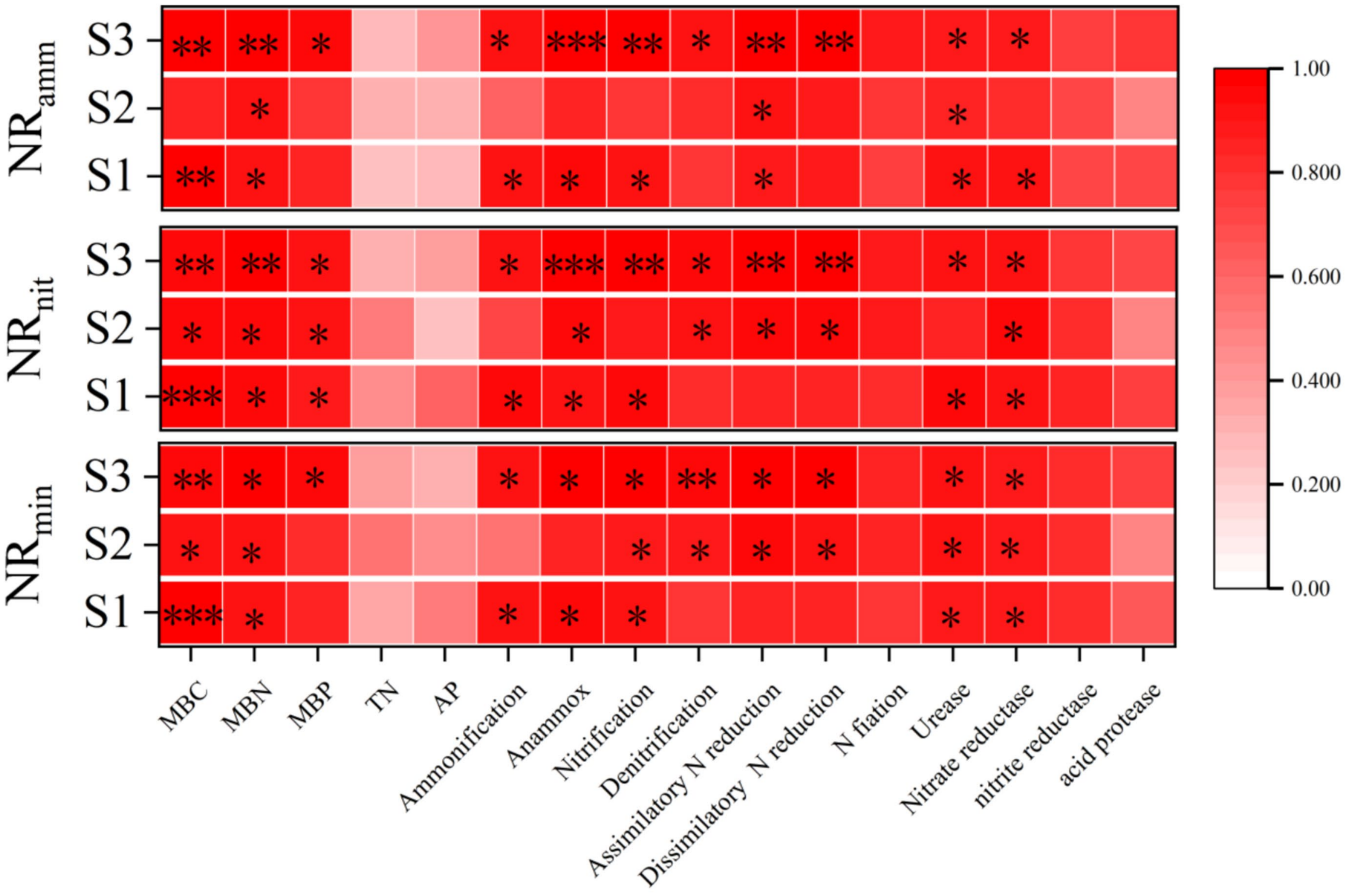
The correlation between net ammonification, net nitrification, and net mineralization in soil and soil environmental factors. The data are all presented as the average values during the sampling period; NR_amm_, NR_nit_, NR_min_ represent net ammonification, net nitrification, and net mineralization in soil, respectively: *p <= 0.05, **p <= 0.01, ***p <= 0.001.

Correlation analysis results (Figure 7) indicated that under the S1 salinity condition, net ammonification was significantly positively correlated with microbial biomass carbon (MBC), microbial biomass nitrogen (MBN), and nitrogen functional genes (NFGs) related to ammonification, anammox, nitrification, and assimilatory nitrate reduction. In addition, it was significantly positively correlated with urease and acid protease activities. Both net nitrification and net mineralization were significantly positively correlated with MBC and MBN, and with ammonification, anammox, and nitrification genes, as well as with urease and acid protease activities. Under the S2 salinity condition, net ammonification was significantly positively correlated only with MBN, ammonification, anammox genes, and urease activity. Net nitrification showed significant positive correlations with MBC, MBN, microbial biomass phosphorus (MBP), anammox, denitrification, assimilatory nitrate reduction, and dissimilatory nitrate reduction genes, along with acid protease activity. Net mineralization was significantly positively correlated with MBC, MBN, nitrification, denitrification, assimilatory and dissimilatory nitrate reduction genes, and also with urease and acid protease activities. Under the S3 salinity condition, mineral nitrogen contents were significantly positively correlated with microbial biomass and all nitrogen functional genes except for nitrogen fixation. Moreover, they were also significantly positively correlated with urease and acid protease activities.

**Figure 8:**
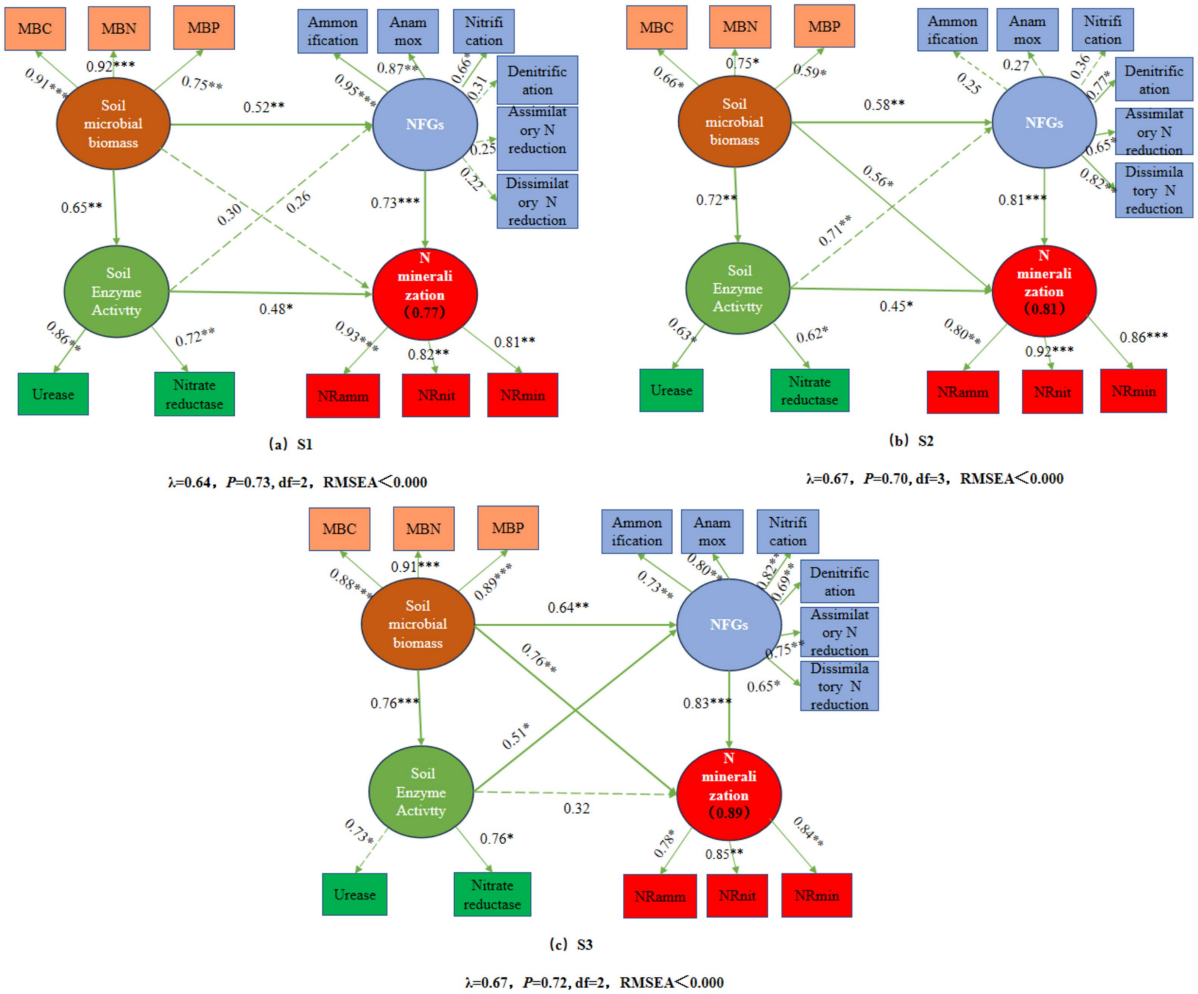
Structural equation modeling. Panels **(a–c)** represent the structural equation modeling (SEM) analyses under salinity levels S1, S2, and S3, respectively. The numbers in the boxes indicate the percentage of variance in the response variables explained by the predictor variables. Solid arrows and dashed arrows represent significant and non-significant relationships, respectively; green arrows indicate positive correlations. The numbers above the arrows represent standardized path coefficients (*p < 0.05, **p < 0.01, ***p < 0.001). *χ*^2^ denotes the chi-square value; *p* represents the probability level; and df indicates the degrees of freedom.

Structural equation modeling (SEM) revealed that soil microbial biomass, nitrogen functional gene (NFG) abundance, and soil enzyme activities exerted significant direct or indirect effects on soil nitrogen mineralization (Figure 8). The final SEMs explained 77, 81, and 89% of the total variation in nitrogen mineralization under S1, S2, and S3 salinity conditions, respectively (Figures 8a–c). Under S1 salinity conditions, the abundance of nitrogen-cycling functional genes and soil enzyme activities exerted positive effects on nitrogen mineralization. Soil microbial biomass may indirectly influence nitrogen mineralization by affecting the abundance of nitrogen-cycling functional genes and soil enzyme activities. Under S2 salinity conditions, soil microbial biomass, nitrogen-cycling functional gene abundance, and soil enzyme activities all showed positive effects on nitrogen mineralization. Under S3 salinity conditions, soil microbial biomass and nitrogen-cycling functional gene abundance had positive effects on nitrogen mineralization, while soil enzyme activity may have indirectly influenced nitrogen mineralization by affecting the abundance of nitrogen-cycling functional genes.

## 4 Discussion

### 4.1 Effects of biochar pyrolysis temperature on soil nitrogen, phosphorus, and microbial biomass

In this study, increasing soil salinity significantly reduced total nitrogen (TN), available phosphorus (AP), and microbial biomass carbon, nitrogen, and phosphorus (MBC, MBN, and MBP), consistent with previous findings (Nur et al., 2025). This was primarily attributed to the osmotic stress and ion toxicity under saline conditions, which inhibited microbial growth and metabolic activity, thereby reducing microbial nutrient transformation and accumulation capacity (Li H. P. et al., 2025; Li W. et al., 2025). Moreover, salinity disrupts soil aggregate structure, impairs aeration and water retention, and consequently constrains nitrogen mineralization, phosphorus release, and microbial biomass formation (Li et al., 2014). Our results further demonstrated that biochar application significantly enhanced microbial biomass and AP content. Under low salinity conditions, biochar produced at low-to-moderate pyrolysis temperatures (300–500 °C) were more effective, whereas under high salinity, high-temperature biochar (700 °C) exhibited the most pronounced enhancement of microbial biomass and available phosphorus. This could be due to the retention of more labile organic carbon and soluble nutrients (e.g., low molecular weight organic acids) in biochar pyrolyzed at 300–500 °C (Wu et al., 2019), which are more readily utilized by microbes under low-salinity conditions, thereby promoting microbial proliferation and phosphorus mobilization (Ye et al., 2025). In contrast, under high salinity, microbes face stronger osmotic and ionic stress. High-temperature biochar (700 °C), characterized by higher surface area, well-developed porosity, and stronger cation exchange capacity (CEC), can effectively adsorb toxic ions such as Na^+^ and Cl^−^, alleviate salt stress, and create a more favorable environment for microbial growth (Zhou et al., 2024; Gao et al., 2024). The lack of significant effect of biochar application on soil TN content in this study may be due to the substantial loss of volatile nitrogen compounds during pyrolysis, resulting in low intrinsic nitrogen content in biochar, which limits its short-term contribution to soil TN (De Oliveira Paiva et al., 2024).

### 4.2 Effects of biochar pyrolysis temperature on nitrogen mineralization in saline soils of varying salinity levels

This study revealed that under low and moderate salinity conditions, the net ammonification rate in the CK (control) treatment was negative during incubation, indicating that NH₄^+^-N consumption exceeded production. However, when soil salinity reached 2.79 dS m^−1^, net ammonification became positive, possibly due to suppressed microbial activity at higher salinities (Andronov et al., 2012; Yan and Marschner, 2012), resulting in mineralization exceeding microbial immobilization. These findings are consistent with Zhou et al. (2020). Co-application of biochar and fertilizer significantly increased net ammonification during the later incubation stages compared to fertilizer alone, which may be attributed to the slow-release effect of biochar on NH₄^+^ and stimulation of ammonifying microbial activity (Song et al., 2014). Moreover, when soil salinity was below 1.92 dS m^−1^, soil net ammonification showed a decreasing trend with increasing biochar pyrolysis temperature. This may be attributed to the structural transformation of biochar at higher pyrolysis temperatures, characterized by enhanced aromaticity and increased structural stability, which in turn alters its nitrogen adsorption and immobilization behavior. Previous studies have demonstrated that low-temperature biochar typically retains more oxygen-containing functional groups (e.g., carboxyl and hydroxyl groups), which contribute to surface charge development and cation exchange capacity. In contrast, high-temperature biochar exhibits greater structural stability and stronger aromatic carbon characteristics, and its adsorption of NH₄^+^-N may be enhanced through physical adsorption and pore-filling mechanisms (Mukherjee et al., 2011). Conversely, under high salinity (2.79 dS m^−1^), high-temperature biochar more effectively enhanced net ammonification, likely due to improved porosity and aeration, facilitating aerobic microbial activity (Zhou et al., 2024; Yang et al., 2022), and its strong ion exchange capacity and alkalinity that mitigate ionic toxicity (An et al., 2023). However, this study did not further characterize the surface functional groups and related properties of the biochar, which may to some extent limit the depth of the mechanistic interpretation. Therefore, future studies should incorporate characterization techniques such as FTIR to systematically analyze the surface chemical properties of biochar, thereby further enhancing the understanding of its regulatory mechanisms in nitrogen transformation processes.

Net nitrification rates were also markedly influenced by both soil salinity and biochar pyrolysis temperature. At low salinity (0.78 dS m^−1^), net nitrification was negative within the first 3 days of incubation across all treatments, possibly due to the slow onset of the nitrification process and strong microbial competition for nitrate under low salt stress (Pang et al., 2024). As salinity increased, net nitrification turned positive in all treatments, reflecting a decline in microbial immobilization under salt stress (Li et al., 2021). Zeng et al. (2013) reported that soil EC1:5 values below 1.13 dS m^−1^ promote nitrification, whereas higher values inhibit it—a trend consistent with our findings at 0.78–2.79 dS m^−1^. Notably, under low-to-moderate salinity, biochar produced at 500 °C resulted in the highest net nitrification, potentially due to its ability to release dissolved organic carbon (DOC), which activates nitrifiers. Under high salinity (2.79 dS m^−1^), the highest net nitrification was observed in the N1C3 treatment, which may be due to: (1) high-temperature biochar’s capacity to retain and gradually release NH₄^+^, providing a sustained substrate for nitrification (Zhang et al., 2020), and (2) its promotion of halophilic and salt-tolerant nitrifiers, shifting microbial communities toward enhanced nitrification (An et al., 2023).

Both soil salinity and biochar pyrolysis temperature significantly affected net nitrogen mineralization. As salinity increased, net N mineralization in CK shifted from negative to positive, indicating that microbial humification processes weakened relative to mineralization (Dong et al., 2025). Across treatments, net mineralization generally declined with increasing salinity, consistent with Chandra et al. (2002), likely due to salinity-induced inhibition of microbial activity, functional diversity, and related enzyme systems (Li et al., 2021). At low salinity (0.78 dS m^−1^), net mineralization increased over time, whereas under moderate to high salinity (≥1.92 dS m^−1^), a rise–fall–rise pattern emerged. This may reflect transient NO₂^−^ accumulation due to inhibited nitrification steps, reducing mineral N content, followed by increased microbial salt tolerance and resumed mineralization in later stages (Zeng et al., 2013; Asghar et al., 2012).

### 4.3 Influence of soil environmental factors on nitrogen mineralization processes

Microorganisms play a central role in soil nutrient cycling. In this study, biochar addition significantly altered microbial biomass, nitrogen cycling gene abundance, and enzyme activities in saline soils of different salinity levels, thus affecting nitrogen mineralization (Ding, 2023). Under low salinity conditions, the application of medium-temperature biochar (500 °C) significantly increased the abundance of genes associated with ammonification, anammox, and nitrification, whereas the abundance of genes related to denitrification, assimilatory N reduction, and dissimilatory nitrate reduction was higher in the low-temperature biochar (300 °C) treatment. This difference may be attributed to the moderate aromaticity of BC500 (H/C = 0.51) and its relatively larger specific surface area and pore volume (Table 2), which favor the formation of a more aerated microenvironment, thereby promoting aerobic processes such as ammonification and nitrification. In contrast, BC300 exhibited higher H/C, O/C, and (O + N)/C ratios, as well as higher contents of N, P, and K, indicating the presence of more labile carbon and nutrients. These characteristics likely stimulated heterotrophic activity and reductive nitrogen transformation processes, including denitrification and dissimilatory nitrate reduction (Wang et al., 2023).

Under moderate and high salinity conditions, the highest abundance of nitrogen-cycling genes was observed in the BC500 and BC700 treatments, respectively. This may be because BC500 possesses relatively high cation exchange capacity (12.8 cmol kg^−1^) and moderate alkalinity (pH 9.0), enabling adsorption of excess Na^+^ and Cl^−^ in soil and alleviation of salt-induced ionic toxicity (Domingues et al., 2020; Gao et al., 2024). Moreover, BC700, characterized by higher aromaticity and greater structural stability, is less susceptible to microbial degradation under high-salinity conditions and can provide long-term physical protection and buffering capacity, thereby shielding microorganisms from direct salt stress (Li et al., 2023). Furthermore, under low and moderate salinity, soil urease and acid protease activities were highest in the BC500 treatment. This response may be attributed to the relatively large specific surface area, moderate organic carbon release capacity, and effective salt-mitigation properties of BC500, which collectively enhanced the activity of enzyme-producing microorganisms and thereby increased enzyme activities (Wu et al., 2019). In contrast, under high salinity conditions, although BC700 exhibited lower CEC, its higher aromaticity, larger surface area, and greater pore volume conferred stronger structural stability. These properties may facilitate the formation of persistent microhabitats, buffer osmotic stress, and protect extracellular enzymes from structural degradation (Li et al., 2023).

Pearson correlation analysis indicated that MBC and MBN were positively correlated with nitrogen mineralization, suggesting that microbial biomass enhances the conversion of organic to inorganic nitrogen across salinity gradients (Shi et al., 2019). Additionally, under low salinity, mineralization was mainly correlated with ammonification, anammox, and nitrification genes; under moderate salinity, with denitrification and both assimilatory and dissimilatory N reduction genes; and under high salinity, with all six gene groups. This suggests a transition from aerobic-dominated to a more diverse and functionally redundant network involving facultative and anaerobic pathways under increasing salinity (Wang et al., 2018). From the perspective of nitrogen cycling, this metabolic shift may indicate an increased risk of nitrogen loss. On the one hand, the denitrification process reduces NO_3_^−^ to N_2_O and N_2_, and N_2_O is a potent greenhouse gas (Setia et al., 2011). On the other hand, under salt stress conditions, insufficient expression of the nosZ gene or incomplete denitrification may lead to the accumulation and enhanced emission of N_2_O (Butterbach-Bahl et al., 2013). In addition, Nitrogen mineralization also showed significant positive correlations with urease and acid protease activities, highlighting the key roles of enzymatic hydrolysis in transforming organic nitrogen into NH₄^+^. Acid protease facilitates nitrogen substrate release, while urease catalyzes urea hydrolysis—both acting as critical steps in the mineralization chain (Sawicka et al., 2020).

Overall, this study demonstrates that under low to moderate salinity conditions, the application of biochar produced at a moderate pyrolysis temperature (500 °C) enhances soil microbial biomass, the abundance of nitrogen-cycling functional genes, and the activities of urease and acid protease—factors closely associated with soil nitrogen mineralization. In contrast, under high salinity conditions, biochar produced at a high pyrolysis temperature (700 °C) exhibits superior performance. These findings partly explain the differential effects of biochar pyrolysis temperature on nitrogen mineralization across soils with varying salinity. However, this study was conducted under laboratory incubation conditions, which cannot fully simulate the complex and dynamic environmental factors present in long-term field settings, such as biochar aging, plant root regulation, and climatic fluctuations, and their integrated effects on soil nitrogen cycling. In addition, the analysis of microbial functional genes was performed at only three sampling time points, representing the early, middle, and late stages of incubation. Although this design captures the stage-specific responses of nitrogen cycling processes under different treatments, soil microbial communities and functional gene expression typically respond rapidly and dynamically to environmental changes. Therefore, the limited temporal resolution may not fully capture short-term fluctuations and transient peaks, thereby constraining the detailed interpretation of microbial response processes.

## 5 Conclusion

To elucidate the effects of biochar produced at different pyrolysis temperatures on nitrogen mineralization in saline soils, an incubation experiment was conducted under gradients of slight, moderate, and severe salinity. The main conclusions are as follows:

(1) The application of biochar significantly enhanced microbial biomass and the activities of urease and acid protease in soils with varying salinity levels. In addition, the relative abundances of key nitrogen cycling functional genes (NFGs), including those involved in ammonification, nitrification, denitrification, and anaerobic ammonium oxidation (anammox), were markedly increased, except for nitrogen fixation genes.
(2) Nitrogen mineralization was significantly affected by the interaction between soil salinity level and biochar pyrolysis temperature. At slight and moderate salinity levels, medium-temperature biochar (500 °C) was more effective in promoting nitrogen mineralization, whereas under severe salinity conditions, high-temperature biochar (700 °C) exhibited superior performance.
(3) Correlation analysis and structural equation modeling (SEM) indicated that: Under slight salinity, medium-temperature biochar (500 °C) facilitated nitrogen mineralization by increasing microbial biomass, urease and acid protease activities, and the abundances of ammonification, anammox, and nitrification genes; Under moderate salinity, its effect was mainly attributed to the enhanced abundances of genes related to denitrification, assimilatory nitrogen reduction, and dissimilatory nitrogen reduction; Under severe salinity, high-temperature biochar (700 °C) significantly promoted nitrogen mineralization by comprehensively increasing microbial biomass, enzyme activities, and the expression of a broad spectrum of nitrogen cycling genes.
(4) Future studies should conduct long-term field experiments to validate the applicability and stability of the results obtained from laboratory incubation under real agricultural ecosystem conditions. In addition, the combined effects of biochar with other soil amendments (e.g., gypsum and organic fertilizers) should be further explored to evaluate their potential synergistic mechanisms and their comprehensive impacts on nitrogen use efficiency and environmental risks.

## Data Availability

The original contributions presented in the study are included in the article/supplementary material, further inquiries can be directed to the corresponding authors.
